# Antipsychotic treatment patterns and cardiometabolic medicine use: current real-world evidence

**DOI:** 10.1017/S2045796026100468

**Published:** 2026-02-11

**Authors:** Ramya Padmavathy Radha Krishnan, Helga Zoega, Nicholas A Buckley, Jacques Eugene Raubenheimer

**Affiliations:** 1 Sydney Pharmacy School, Faculty of Medicine and Health, The University of Sydney, Sydney, NSW, Australia; 2 Centre of Public Health Sciences, Faculty of Medicine, University of Iceland, Reykjavik, Iceland

**Keywords:** antipsychotic, cardiometabolic medicine use, cardiovascular and metabolic adverse effects, population-based, real-world evidence

## Abstract

**Aims:**

Off-label use of antipsychotics, often at low doses, is increasing. Exploring the link between individual antipsychotic treatment patterns, including low-dose continuous use, and cardiometabolic health is crucial to prevent long-term morbidity and mortality. The current retrospective study examined the prevalence of cardiometabolic medicine use among antipsychotic-users, and its association with their past antipsychotic treatment patterns.

**Methods:**

Using a 10% sample of the Australian national medicine dispensing claims data from 2022, we identified individuals aged 15–64 years with ≥2 antipsychotic dispensings (antipsychotic-users) and non-users. We extracted their past 5-year antipsychotic treatment patterns (dose, duration and use of multiple agents). Using Poisson regression and accounting for age and sex, we calculated adjusted prevalence ratios (aPR) and 95% confidence intervals (CI) for cardiometabolic medicine use (anti-diabetics, antihypertensives, lipid modifiers, anti-thrombotics) among antipsychotic-users versus non-users. We applied unsupervised hierarchical clustering analysis to identify common antipsychotic-cardiometabolic co-dispensing.

**Results:**

Use of any cardiometabolic medicine was more prevalent among antipsychotic-users (35.8%, *n* = 28,345) than non-users (26%, *n* = 1,106,610) yielding an aPR of 1.30 (CI 1.28–1.33). aPRs for the use of anti-diabetics, lipid modifiers and antihypertensives were the highest among the younger age groups between 20 and 49 years and among women. Clustering analysis revealed increased co-dispensing of antipsychotics and anti-diabetics including sulfonylureas, statins, platelet aggregation inhibitors and beta blockers. The prevalence of cardiometabolic medicine use was associated with higher antipsychotic doses (23–54%), treatment duration (12–37%) and use of multiple agents (51%) compared with non-users. However, the prevalence of cardiometabolic medicine use for continuous (≥1 year) low-dose use of aripiprazole, asenapine, brexpiprazole, chlorpromazine, lurasidone, olanzapine, periciazine and quetiapine was also elevated (13–43%).

**Conclusions:**

Use of cardiometabolic medicines is increased among people on long-term antipsychotic treatment. These results highlight the need for active monitoring for cardiometabolic adverse effects, with antipsychotic cessation where possible, or timely interventions to limit morbidity.

## Introduction

Antipsychotics are widely prescribed for both psychotic and non-psychotic conditions, with growing concerns about their cardiometabolic impact (Carton *et al.*, 2015). They are linked to metabolic dysregulation (weight gain, hyperglycaemia and dyslipidaemia), increasing risks of developing type 2 diabetes, metabolic syndrome and cardiovascular sequelae and imposing significant health and economic burdens (De Hert *et al.*, 2012). The aetiology of these adverse effects in schizophrenia is complex and multifactorial, with increased prevalence of metabolic and other physical abnormalities before pharmacotherapy, further exacerbated by antipsychotic use (Kirkpatrick *et al.*, 2014). The antipsychotic agent used and the intensity of exposure may also play a role (Leucht *et al.*, 2013; Correll *et al.*, 2015). High-risk antipsychotics include olanzapine and clozapine, and to some extent, quetiapine and risperidone, while aripiprazole and ziprasidone cause minimal effects (Daumit *et al.*, 2008; Reynolds and Kirk, 2010; Rummel-Kluge *et al.*, 2010; Pillinger *et al.*, 2020). Individual characteristics (genetics, lifestyle and other health conditions) likely modify the risks for these effects.

Off-label antipsychotic use, often at low doses, is increasing globally, and can range from 40% to 75% of antipsychotic prescriptions (Carton *et al.*, 2015; Hálfdánarson *et al.*, 2017; Radha Krishnan *et al.*, 2023). Common indications include anxiety disorders, depression, insomnia and dementia, often reflecting limited availability/tolerability of approved treatments, psychiatric comorbidities or persisting clinical challenges (Carton *et al.*, 2015; Bell and Richards, 2021). However, evidence on antipsychotic effectiveness and tolerability is limited, especially with long-term use due to the chronic nature of these disorders (Patten *et al.*, 2012; Verdoux, 2025). For example, antipsychotics reduced irritability and self-injury but not aggression in participants with autism, but only in short-term (<6 months) trials (Iffland *et al.*, 2023). Similarly, they displayed minimal or no effects in individuals with borderline personality disorder (Stoffers-Winterling *et al.*, 2022). Moreover, clinical guidelines for many non-psychotic disorders recommend against antipsychotic use (e.g., insomnia) or advise cautious use in treatment-refractory individuals (e.g., anxiety disorders and depression) (Bandelow *et al.*, 2022; National Institute for Health and Care Excellence, 2022; Riemann *et al.*, 2023).

Metabolic effects are reported with low-dose antipsychotic use in non-psychotic conditions, primarily in short-term trials with standardised regimens that exclude high-risk patients (Linton *et al.*, 2013; Alfageh *et al.*, 2019; Iasevoli *et al.*, 2020; Stogios *et al.*, 2022). In contrast, real-world use is often long-term and characterised by psychiatric comorbidity and concomitant psychotropic therapies. Additionally, treatment effects demonstrated in trials may appear greater than those observed in real-world settings (Efthimiou *et al.*, 2024). Thus, evidence from population-based studies can provide valuable insights into real-world cardiometabolic health of antipsychotic-users.

We conducted the current study to examine cardiometabolic medicine use among antipsychotic-users compared with non-users, specifically the prevalence of medicines used to manage hypertension, hyperglycaemia, dyslipidaemia and thrombosis. We then examined the influence of their past antipsychotic treatment patterns, including long-term low-dose use, on this cardiometabolic medicine use. This approach reflects real-world health accurately while still capturing the accumulated effects of long-term use.

## Methods

This population-based retrospective study followed the REporting of studies Conducted using Observational Routinely-collected Data–PharmacoEpidemiology (RECORD-PE) guidelines (Supplementary material) (Langan *et al.*, 2018).

### Study population

The Pharmaceutical Benefits Scheme (PBS), an Australian government initiative under its publicly funded Medicare system, provides subsidised medicines to citizens, permanent residents and visitors from countries with healthcare agreements (Mellish *et al.*, 2015). It includes community and private hospital-dispensed medicines, while excluding dispensings to public hospital in-patients, private prescriptions, over-the-counter medicines and to veterans under the Repatriation PBS (Page *et al.*, 2015). These data are increasingly used in pharmacoepidemiologic research, especially for psychotropics and cardiovascular medicines (Pearson *et al.*, 2021). A deidentified, nationally representative 10% random sample of PBS dispensing claims is available for research purposes through Services Australia, with individuals retained over time to facilitate longitudinal analyses (Mellish *et al.*, 2015). We used this 10% sample (updated to March 2025), with the study population consisting of individuals with any dispensing claim during 1 Jan to 31 December 2022. We retrieved their prior antipsychotic dispensing claims (2017–2022) to establish antipsychotic treatment patterns, and from 2005 to identify non-users, as detailed below.

### Antipsychotic exposure and treatment patterns

We selected individuals with ≥2 antipsychotic dispensings in 2022 within a treatment episode (described below) as antipsychotic-users. We included all antipsychotics (except lithium) subsidised by PBS, including oral and long-acting injections (Supplementary eTable 1). Antipsychotic non-users were individuals dispensed any other medicine in 2022, and without prior antipsychotic dispensings from 2005 onwards. We restricted the analyses to individuals aged between 15 and 64 years, as children and older individuals may differ in their antipsychotic and/or cardiometabolic medicine use. Adolescents between 15 and 19 years were included due to high rates of off-label antipsychotic prescribing (Karanges *et al.*, 2014; Rao *et al.*, 2024) and their propensity to develop cardiometabolic perturbations with prolonged treatment (Radha Krishnan *et al.*, 2025).

**Table 1.** Prevalence of cardiometabolic medicine use

	Individuals, n	Cardiometabolic medicine use, *n* (%)	Standardised prevalence, % (95% CI)	Adjusted prevalence ratio (95% CI)
**Lipid-modifying agent use**				
Non-users	1,016,610	126,563 (12.5)	10.7 (10.7−10.8)	Ref
Antipsychotic-users	28,345	5,672 (20.0)	16.4 (16.0−16.9)	1.5 (1.5−1.6)
Antipsychotic polypharmacy	11,507	2,567 (22.3)	19.1 (18.4−19.9)	1.8 (1.8−1.9)
Low-dose continuous therapy	6,900	1,181 (17.1)	14.3 (13.5−15.1)	1.4 (1.3−1.5)
**Anti-diabetic medicine use**				
Non-users	1,016,610	71,345 (7.0)	6.3 (6.3−6.4)	Ref
Antipsychotic-users	28,345	4,419 (15.6)	14.2 (13.8 − 14.6)	2.1 (2.1−2.2)
Antipsychotic polypharmacy	11,507	2,461 (21.4)	20.6 (19.7−21.5)	2.9 (2.8−3.0)
Low-dose continuous therapy	6,900	793 (11.5)	10.3 (9.5−11.0)	1.6 (1.5−1.7)
**Antihypertensive medicine use**				
Non-users	1,016,610	172,408 (17.0)	14.8 (14.7−14.9)	Ref
Antipsychotic-users	28,345	5,304 (18.7)	15.7 (15.3−16.2)	1.04 (1.01−1.1)
Antipsychotic polypharmacy	11,507	2,154 (18.7)	16.4 (15.7−17.1)	1.1 (1.1−1.6)
Low-dose continuous therapy	6,900	1,287 (18.7)	16.0 (15.1−16.9)	1.08 (1.0−1.6)
**Anti-thrombotic medicine use**				
Non-users	1,016,610	33,061 (3.3)	2.9 (2.9−2.9)	Ref
Antipsychotic-users	28,345	1,380 (4.9)	4.2 (4.0−4.5)	1.4 (1.4−1.5)
Antipsychotic polypharmacy	11,507	555 (4.8)	4.4 (4.0−4.8)	1.4 (1.3−1.6)
Low-dose continuous therapy	6,900	334 (4.8)	4.2 (3.8−4.7)	1.46 (1.3−1.6)
**Any cardiometabolic medicine use**				
Non-users	1,016,610	264,697 (26.0)	23.1 (23.0−23.2)	Ref
Antipsychotic-users	28,345	10,158 (35.8)	31.3 (30.7−31.9)	1.3 (1.3−1.3)
Antipsychotic polypharmacy	11,507	4,664 (40.5)	37.3 (36.2−38.5)	1.5 (1.5−1.6)
Low-dose continuous therapy	6,900	2,269 (32.9)	28.7 (27.5−29.9)	1.2 (1.2−1.3)

Crude prevalence proportions, age-/sex-standardised prevalence proportions and adjusted prevalence ratios (aPR, adjusted for age and sex) with 95% confidence intervals (CI) for cardiometabolic medicine use are given, comparing antipsychotic-users with non-users.

PBS claims lack details on daily dose or treatment duration. We estimated antipsychotic treatment patterns using an individualised dispensing patterns method, which estimates treatment duration per individual based on the quantity dispensed and time between dispensings (Bharat *et al.*, 2023). For each unit (tablet or injection), we used data from individuals with ≥2 dispensings (<180 days apart) to estimate days covered per unit. Using the 80th percentile of this estimate, we calculated duration of the first dispensing based on units dispensed per individual. This cut-off is widely used in literature to estimate dispensing intervals in real-world data (Pottegård and Hallas, 2013) and further recommended by Bharat *et al.* (2023). Subsequently, we calculated dispensing duration based on the prior three dispensings per individual. Treatment episodes were continuous exposure periods with successive dispensings within the estimated dispensing duration, allowing a 15-day grace period for delays in filling prescriptions. Treatment duration per episode was the time from first to last dispensing plus the estimated dispensing duration.

We calculated each individual’s cumulative treatment duration across all episodes and categorised as follows: short-term (≤6 months), medium-term (>6 months–1 year), long-term (>1–3 years) and ultra-long-term (>3 years). We converted antipsychotic doses to olanzapine equivalents (OE) using the defined daily dose method (Leucht *et al.*, 2016). The daily dose per episode was the total OE for all dispensings divided by the treatment duration. We categorised each individual’s average daily dose across all episodes as: low (>0–5 mg/day), moderate (>5–10 mg/day), high (>10–20 mg/day) and very high (>20 mg/day), based on recommended dosing (Gardner *et al.*, 2010). We applied a 30% dose reduction for individuals aged 15–19 years to reflect real-world prescribing (Gardner *et al.*, 2010). We allowed overlaps between formulations or multiple agents for overall antipsychotic analyses, and between formulations of the same antipsychotic agent for agent-specific analyses. We included individuals using a single agent in a treatment episode in the respective monotherapy group. Individuals could contribute to multiple monotherapy groups from different treatment episodes during the study period. We defined antipsychotic polypharmacy as overlapping use of ≥2 agents for >30 days.

### Cardiometabolic medicine use

We identified individuals with ≥1 dispensing for cardiometabolic medicines in 2022 among antipsychotic-users and non-users, and included anti-diabetics, lipid-modifying agents, anti-hypertensives and anti-thrombotics (anti-coagulants, anti-platelets and thrombolytics) as listed in the *Australian Medicines Handbook* (*AMH*, Supplementary eTable 2) (AMH Pty Ltd, 2025). Spironolactone and eplerenone, sometimes prescribed for resistant hypertension, are classified under heart failure in the *AMH* and were excluded. We also excluded propranolol, clonidine and prazosin due to their widespread use for non-cardiovascular diseases. We counted combination formulations for both agents.

### Statistical analysis

We used descriptive statistics to summarise the data and compared baseline characteristics between groups using standardised mean difference (SMD). We calculated age in 2022 and stratified into 5-year age groups. We estimated the crude prevalence percentage of cardiometabolic medicine use as the proportion of individuals in 2022 with ≥1 dispensing for cardiometabolic medicines. We derived age- and sex-standardised prevalence estimates using the 2022 Australian resident population (Australian Bureau of Statistics, 2022). We calculated prevalence ratios between antipsychotic-users and non-users by age and sex strata, displayed in forest plots. We used Poisson regression to estimate adjusted prevalence ratios (aPR) and 95% confidence intervals (CI) for cardiometabolic medicine use, accounting for age (as a continuous variable) and sex, comparing overall antipsychotic-users and subgroups with non-users. We further identified antipsychotic-users dispensed low-dose antipsychotics (≤5 mg/day OE) for ≥1 year and compared with non-users. We also calculated aPRs for each individual antipsychotic-cardiometabolic medicine pair and by medicine class and ran unsupervised hierarchical clustering analyses (more details in Supplementary material). This method can help identify clinically meaningful antipsychotic-cardiometabolic medicine pairs that are preferentially co-dispensed, thereby revealing clusters of medicine combinations that reflect distinct dispensing patterns and metabolic risk profiles. The results of these analyses are displayed both individually and in a semi-structured heatmap.

Using the 80th percentile of the estimated days covered per unit may introduce exposure misclassification; hence, we conducted sensitivity analyses by selecting percentiles between 70 and 90 to estimate the cumulative treatment duration and average daily dose for the antipsychotic treatment patterns. We used the SAS/STAT version 15.1 (Windows version 9.4) for data extraction and analyses.

## Results

### Cohort characteristics

There were 1,741,076 individuals in the study population in 2022, and the final cohort included 28,345 antipsychotic-users and 1,016,610 non-users aged between 15 and 64 years (Supplementary eFig. 1). Mean age in 2022 was 42.6 (SD 13.1) years among antipsychotic-users and 41.2 (14.2) years among non-users (SMD 0.1), and the proportion of men was 52.5% and 43.8%, respectively (SMD 0.1).

### Prevalence of cardiometabolic medicine use

Table 1 presents crude and standardised prevalence proportions and aPR with 95% CI for cardiometabolic medicine use. Both crude and standardised prevalence rates for the use of all four medicine classes were higher in antipsychotic-users than non-users. After adjusting for age and sex in the regression model, the prevalence of anti-diabetic use in antipsychotic-users was higher by 112%, lipid-modifying agents by 52%, antihypertensives by 4% and anti-thrombotic use by 44% compared with non-users. Overall, any cardiometabolic medicine use was 30% more prevalent among antipsychotic-users than non-users.

**Figure 1.**
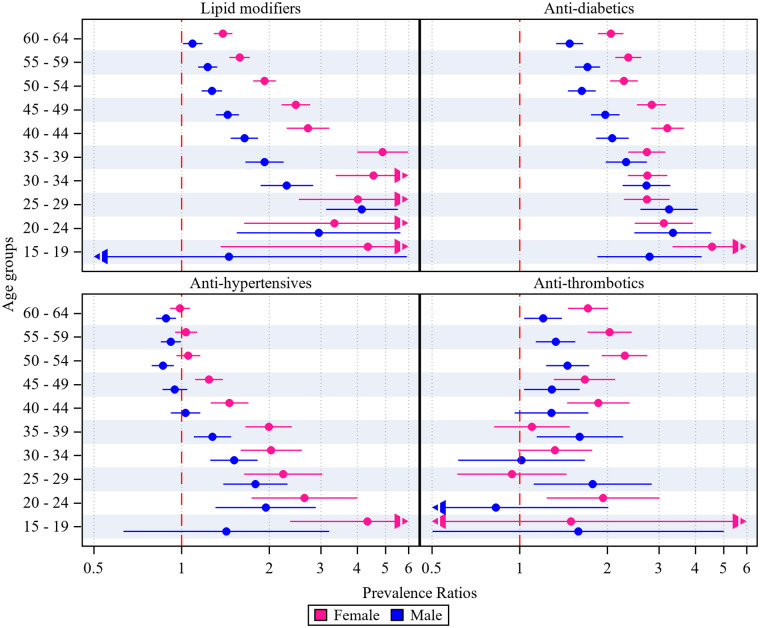
Age and sex stratified prevalence ratios for cardiometabolic medicine use, comparing antipsychotic-users with non-users for each age and sex stratum, with the upper and lower confidence limits displayed as bars.

Figure 1 displays age- and sex-stratified prevalence ratios comparing antipsychotic-users with non-users for cardiometabolic medicine use. Ratios were higher in women and younger age groups for the use of lipid-modifying agents, anti-diabetics and antihypertensives, while the ratios for anti-thrombotic use were more variable.

**Figure 2.**
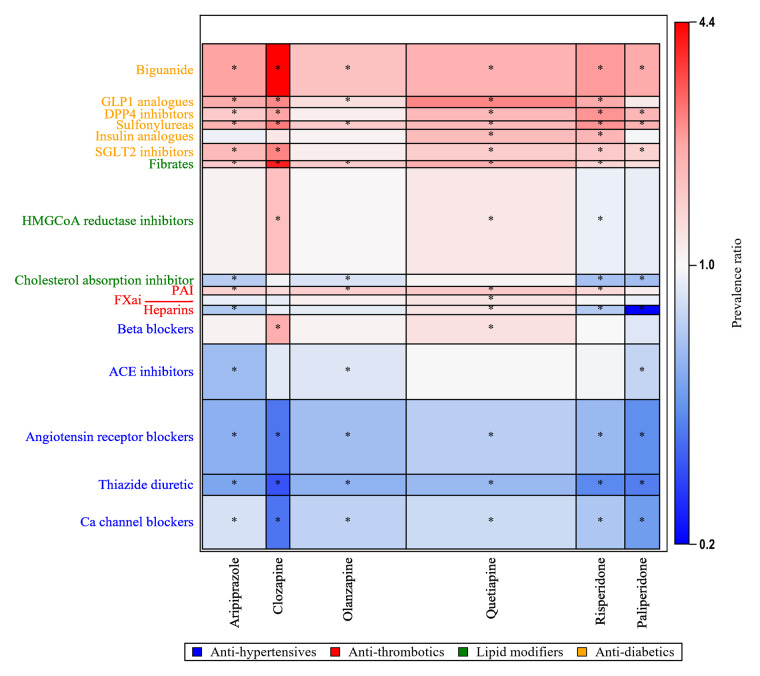
Semi-structured heatmap of the log-transformed prevalence ratios (aPR, adjusted for age and sex) comparing antipsychotic-users with non-users for antipsychotic-cardiometabolic medicine class pairwise combinations. The actual aPR values are displayed in the colour legend for interpretability. aPR values < 1 (blue gradient) are prevalent among non-users and values > 1 (red gradient) are prevalent among antipsychotic-users. Significant 95% confidence intervals are indicated by an asterisk (*). The size of each cell indicates the percentage of individuals using that combination. The position of the antipsychotic agents and cardiometabolic medicine classes are structured based on the clustering analysis. Some cardiometabolic medicine classes-antipsychotic pairs are not shown here as they have very low number of users.

Unsupervised hierarchical clustering analyses used the aPRs for antipsychotic-cardiometabolic medicine pairwise combinations in antipsychotic-users compared with non-users (Supplementary eFig. 2). It revealed broader, data-driven patterns of co-dispensing by grouping medications (both antipsychotics and cardiometabolic medicines) with similar co-dispensing profiles, offering a complementary perspective to the prevalence ratio estimates. These co-dispensing patterns reflect cardiometabolic comorbidity management among antipsychotic-users in routine practice. At the base level, cardiometabolic medicines grouped into four primary clusters. The first two clusters, located towards the bottom of the heatmap, were composed predominantly of antihypertensives and a few anti-thrombotics (e.g., enoxaparin, ticagrelor and clopidogrel) that displayed relatively lower prevalence of co-dispensing among antipsychotic-users. The remaining two clusters contained primarily anti-diabetics and lipid-modifying agents, several with higher prevalence among antipsychotic-users. Antipsychotics clustered into two groups, with the first comprised of agents less commonly used (amisulpride, asenapine, ziprasidone, flupentixol and haloperidol) but with higher prevalence of cardiometabolic medicines including sulfonylureas, aspirin and a few antihypertensives (labetalol, carvedilol, amiloride). Figure 2 presents a semi-structured heatmap of cardiometabolic medicine groups based on their Anatomical Therapeutic Chemical class, and antipsychotic agents used by >5% of the individuals. All the anti-diabetics (including the older-generation sulfonylureas) displayed increased prevalence, along with statins, platelet aggregation inhibitors and beta blockers.

### Influence of treatment patterns

Antipsychotic treatment patterns among antipsychotic-users (2017–2022) are summarised in Supplementary eTable 3. The average daily dose in OE was 9.6 (SD 8.4) mg/day, with 28.2% taking moderate doses and 34.3% taking ≤5 mg/day. Median treatment duration was 1,420 (IQR 592–1,976) days (or 3.9 years), and 59.7% were in the ultra-long-term duration category. Antipsychotic polypharmacy was prescribed to 32.5% of antipsychotic-users. There were 9,082 antipsychotic-users (27.8%) dispensed low-dose antipsychotics for ≥1 year.

**Figure 3.**
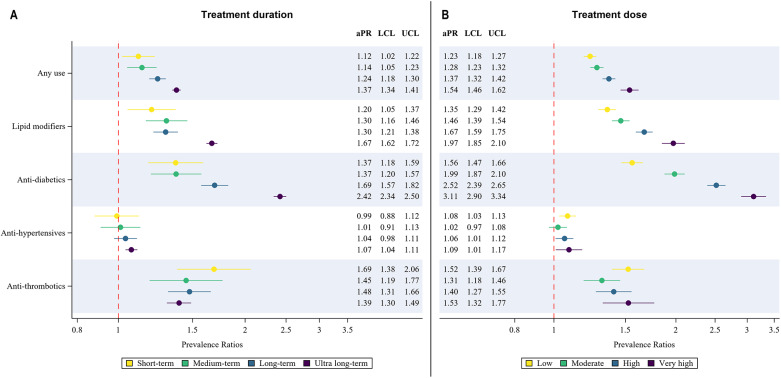
Prevalence ratios (adjusted for age and sex) for cardiometabolic medicine use comparing antipsychotic-users with non-users for A) treatment duration and B) average daily dose categories. Upper and lower confidence limits are displayed as bars. aPR: adjusted prevalence ratios, LCL: lower confidence limit, UCL: upper confidence limit.

The aPRs for cardiometabolic medicine use categorised by treatment dose and duration are displayed in Fig. 3a and 3b. Prevalence of any cardiometabolic medicine use, antihypertensive, anti-diabetic and lipid-modifying agent use showed a dose and duration-dependent increase among antipsychotic-users versus non-users. Anti-thrombotic use was higher in the short-term treatment and low-dose categories, though the aPRs for all other categories were significantly higher than non-users.

Antipsychotic polypharmacy significantly increased the prevalence of all four types of cardiometabolic medicine use compared with non-users (Table 1), with a 51% increase in any cardiometabolic medicine use. We observed a similar increased prevalence of all four cardiometabolic medicine types in the subset of antipsychotic-users with low-dose continuous antipsychotic treatment, with a 24% increase for any cardiometabolic medicine use, as given in Table 1. We analysed low-dose continuous use by specific antipsychotics and identified quetiapine-users as having higher prevalence (11–54%) of all four types of cardiometabolic medicine use versus non-users (Supplementary eTable 4). Low-dose continuous chlorpromazine and periciazine-users had increased prevalence of any cardiometabolic medicine use, lipid-modifying agent, anti-diabetic and anti-thrombotic use, while antihypertensive use trended towards significance.

**Figure 4.**
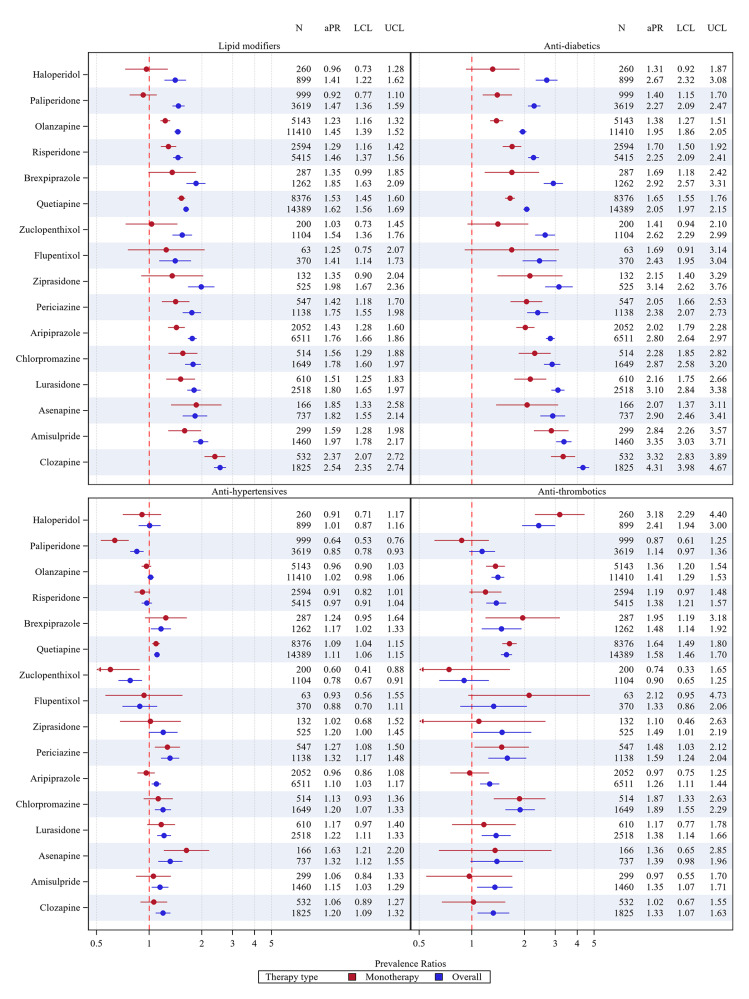
Prevalence ratios (adjusted for age and sex) for cardiometabolic medicine use comparing antipsychotic-users with non-users for each antipsychotic agent, with the upper and lower confidence limits as bars. aPR: adjusted prevalence ratios, LCL: lower confidence limit, UCL: upper confidence limit.

**Figure 5.**
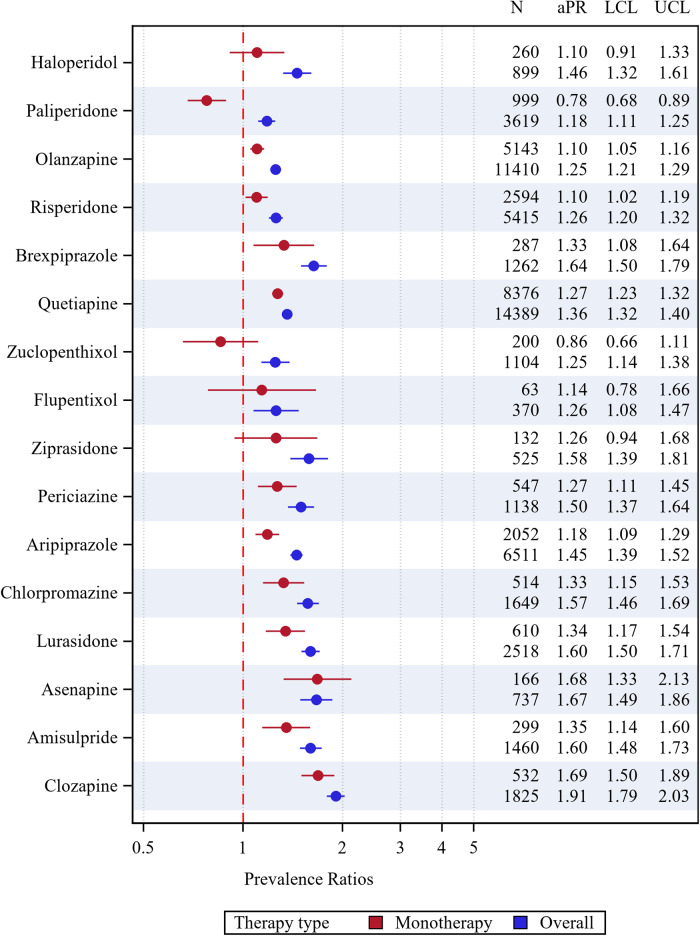
Prevalence ratios (adjusted for age and sex) for any cardiometabolic medicine use comparing antipsychotic-users with non-users for each antipsychotic agent, with the upper and lower confidence limits as bars. aPR: adjusted prevalence ratios, LCL: lower confidence limit, UCL: upper confidence limit.

The aPRs for cardiometabolic medicine use for antipsychotic agent monotherapy and overall, compared with non-users, are presented in Fig. 4 (by type) and Fig. 5 (overall). Users of quetiapine and periciazine displayed increased prevalence of all four cardiometabolic medicine types, while users of asenapine displayed increased prevalence of antihypertensive, anti-diabetic and lipid-modifying agent use. Most antipsychotic monotherapy users had increased prevalence of lipid-modifying agent and anti-diabetic use, though some agents (flupentixol, ziprasidone, haloperidol, zuclopenthixol) had wide confidence intervals. Users of haloperidol, olanzapine, quetiapine, brexpiprazole, periciazine and chlorpromazine had significantly higher prevalence of anti-thrombotic use versus non-users. Ratios for any cardiometabolic medicine use were the highest for clozapine, amisulpride, asenapine, chlorpromazine, brexpiprazole and lurasidone (Fig. 5).

Results of the sensitivity analyses are displayed in Supplementary eTable 5 (treatment dose) and eTable 6 (treatment duration). Varying the percentiles between 70 and 90 had minimal impact on the aPR estimates for all cardiometabolic medicines use.

## Discussion

This study highlights increased cardiometabolic medicine use among individuals using antipsychotics in Australia in 2022. Prevalence ratios for cardiometabolic medicine use in antipsychotic-users versus non-users, adjusted for age and sex, were higher for medicines dispensed to manage hyperglycaemia, dyslipidaemia, hypertension and thrombosis. Younger individuals using antipsychotics had increased prevalence ratios for use of all four types of cardiometabolic medicines, suggesting significantly earlier onset of cardiometabolic perturbations. Prevalence ratios increased with higher exposure intensity and use of antipsychotic polypharmacy, though low-dose continuous use for ≥1 year also displayed increased prevalence. Quetiapine and periciazine users had increased co-dispensing of all four types of cardiometabolic medicines, while paliperidone users had lower use of lipid-modifiers, antihypertensives and anti-thrombotics. These patterns of use may reflect elevated cardiometabolic disease.

Individuals with psychotic disorders have a 69% increased risk of developing two or more chronic physical health conditions (multimorbidity) apart from psychiatric comorbidities (Rodrigues *et al.*, 2021). Diabetes, hypertension, stroke and ischaemic heart disease are among the top 10 comorbidities (De Hert *et al.*, 2011; Bendayan *et al.*, 2022). Multimorbidity contributes substantially to premature mortality and disability in schizophrenia, compounded by cardiometabolic adverse effects of antipsychotics (De Hert *et al.*, 2011; Halstead *et al.*, 2024). Moreover, we observed early cardiometabolic medicine use in younger individuals, similar to previous literature (De Hert *et al.*, 2011, 2012; Halstead *et al.*, 2024). This exposes younger adults to longer durations of compromised physical health which may further intensify over time, widening health disparities with age (De Hert *et al.*, 2011; Halstead *et al.*, 2024). Using cardiometabolic medicines as a proxy may underestimate cardiometabolic disease burden due to infrequent metabolic monitoring, underdiagnosis, suboptimal prescribing and fragmented healthcare services in individuals with schizophrenia (Mitchell *et al.*, 2012; Halstead *et al.*, 2024). Our findings emphasise the need for structured metabolic monitoring, especially in younger individuals, to address cardiometabolic disturbances.

We undertook unsupervised hierarchical clustering analyses to explore distinct patterns of cardiometabolic medicine use across different antipsychotics. We identified medicines co-dispensed with specific antipsychotic agents, even if at different prevalence ratios. This identified data-driven patterns of metabolic risk profiles and their management in practice, while revealing co-treatments of concern. To our knowledge, this is the first comprehensive study of cardiometabolic medicine utilisation among antipsychotic-users in Australia. A major issue with combination treatments is the risk of drug interactions, leading to harmful effects. Physiologically significant interactions in antipsychotic-users include potentiation of diabetes development with statins, compounded effect on weight gain with sulfonylureas, and the potential risk of acute pancreatitis with GLP1 analogues and DPP4 inhibitors, while risks associated with many other medicine classes remain poorly elucidated (Buzea *et al.*, 2022). Identifying these clusters of concurrent medicine use can inform treatment strategies and aid pharmacovigilance.

Off-label antipsychotic prescribing often occurs at lower doses than the recommended therapeutic range (Radha Krishnan *et al.*, 2023). Thus, low doses are sometimes used as a proxy for off-label antipsychotic use in database studies with limited diagnostic details (Højlund *et al.*, 2023). Using this principle, we demonstrated the association of long-term low-dose antipsychotic use (without dose titration) with increased prevalence of cardiometabolic medicine use versus non-users. Quetiapine, the most common agent with long-term low-dose use, was associated with increased prevalence of all four cardiometabolic medicine types compared with non-users (aPRs between 1.11 and 1.54, Supplementary eTable 4). These results are similar to recent studies in literature reporting the association of low-dose quetiapine with abnormalities in weight, glucose regulation and lipid markers (Højlund *et al.*, 2023; Sonim *et al.*, 2025). Thus, clinicians need to consider these cardiometabolic risks while prescribing antipsychotics off-label, avoiding use where possible.

Previous studies have classified antipsychotic agents as high-risk (clozapine, olanzapine, chlorpromazine), moderate-risk (quetiapine, risperidone, paliperidone) and low-risk (amisulpride, aripiprazole, asenapine, lurasidone, ziprasidone) for causing metabolic effects (De Hert *et al.*, 2012). However, this is largely based on short-term clinical trials, with limited data for newer antipsychotics including asenapine, lurasidone, paliperidone and brexpiprazole (De Hert *et al.*, 2012). In contrast, we investigated real-world patterns, with nearly 85% of individuals using antipsychotics >1 year. We observed increased cardiometabolic medicine use among antipsychotic-users including the newer agents, similar to a recent network meta-analysis that analysed the metabolic effects of 31 antipsychotics in mid-to-long term trials (Burschinski *et al.*, 2023). Moreover, almost all antipsychotics cause weight gain to an extent, which is a major risk factor for cardiometabolic disease (Pillinger *et al.*, 2020; Burschinski *et al.*, 2023). An unexpected result in our study was the lower-than-expected prevalence ratios for cardiometabolic medicine use among olanzapine-users, which could be explained by reverse causation, where baseline patient-specific risk factors influence antipsychotic choice (Sattar and Preiss, 2017). Agents like aripiprazole may be preferred for high-risk individuals over other obesogenic agents (De Hert *et al.*, 2012; Leucht *et al.*, 2013). Thus, it is essential that clinicians assess individual risk before prescribing antipsychotics to balance treatment benefits with the risk of potential harms, and revisit treatment regimens at regular intervals.

### Strengths

A key strength of this study is its use of recent health data, providing insight into the cardiometabolic status of individuals actively receiving antipsychotic treatment in 2022. Additionally, the PBS dataset has national whole-population coverage, unlike some other international healthcare schemes like the USA-based Medicare and Medicaid (Kemp *et al.*, 2012). Unlike prospective studies that may often exclude individuals with complex or long-term treatments, our approach captures real-world effects associated with cumulative antipsychotic use. By focusing on real-world treatment patterns rather than the standardised dosing protocols in controlled studies, our study reflects the current real-world context where dose adjustments, polypharmacy and long-term use is common. Finally, this study addresses the gap in knowledge on the safety of long-term, low-dose antipsychotic use, which has become increasingly common in real-world practice.

### Limitations

This study has several limitations, most notably the absence of clinical diagnostic information. Data for other confounders (baseline cardiometabolic risk, socioeconomic conditions and lifestyle behaviours) that may influence antipsychotic treatment patterns and cardiometabolic medicine use were not available. Due to these reasons and being an observational study, we cannot establish causality. Although we applied standardised methods to estimate treatment intensity and conducted sensitivity analyses, exposure misclassification is possible, as the PBS dispensing data lack information on the supply duration or daily dose. Moreover, we calculated a dose reduction of 30% for adolescents (15–19 years old) based on prior literature, which might introduce bias. Fourth, treatment adherence is a problem faced by long-term studies (Correll *et al.*, 2018). We selected individuals with at least two antipsychotic dispensings to include persistent users, but individuals may not adhere to the provided treatment. Fifth, the long-term treatment duration may introduce survivor bias, where people who experience early metabolic effects or weight gain (unlikely within one dispensing period) may discontinue treatment, thus underestimating cardiometabolic medicine use. By including only those individuals with any dispensing in 2022, we might have excluded healthier individuals not using any medicines. Moreover, we did not investigate concurrent use of other medicines with possible metabolic impacts, such as serotonin reuptake inhibitors or valproate, which could potentially confound the observed associations (De Hert *et al.*, 2011). Finally, while the PBS 10% sample is representative of the Australian population accessing subsidised medicines, its generalisability to the global mental health population is uncertain.

### Conclusions and implications

This study highlights increased cardiometabolic medicine use associated with antipsychotics especially among younger individuals, prolonged treatment durations, higher doses and polypharmacy. Clustering analyses revealed distinct patterns of concurrent cardiometabolic medicine and antipsychotic use, indicating potential differences in treatment needs across agents. Metabolic perturbations were evident even among users of low-dose antipsychotics, which may be a potential risk overlooked in clinical practice.

Our findings reinforce the need for cautious prescribing across both psychotic and off-label indications. Clinicians need to carefully weigh potential benefits against harms, particularly at low doses and long-term. Other recommendations include avoiding routine use of antipsychotics for indications such as insomnia or anxiety, when safer alternatives are available (e.g., behavioural therapy), and if required, using agents with lower metabolic liability with ongoing regular review. Shared decision-making and patient education regarding cardiometabolic risks may facilitate the incorporation of lifestyle interventions including diet, exercise and smoking cessation.

Regular metabolic monitoring even for off-label use may mitigate these risks and aid early intervention. Currently, country-specific guidelines differ on the frequency, but generally recommend monitoring at baseline, weeks 6 and 12 for BMI, waist circumference, fasting glucose and lipid markers for individuals with schizophrenia, followed by annual checks for those with normal values (American Diabetes Association *et al*., 2004; De Hert *et al.*, 2009; Galletly *et al.*, 2016). Based on our findings for long-term use and to enable early intervention strategies, we recommend checking BMI, waist circumference and blood pressure at every visit, and bi-yearly fasting glucose and lipid profile monitoring, along with annual electrocardiograph for individuals with baseline risk factors. Clinicians can implement similar monitoring protocols among off-label antipsychotic-users.

## Supporting information

10.1017/S2045796026100468.sm001 Radha Krishnan et al. supplementary material Radha Krishnan et al. supplementary material

## Data Availability

Data were obtained under license from Services Australia of the Australian government. Due to data protection regulations, the data will not be shared.

## Supplementary material

The supplementary material for this article can be found at https://doi.org/10.1017/S2045796026100468.
