# Supplementary material for: Antipsychotic treatment patterns and cardiometabolic medicine use: current real-world evidence
Source: Epidemiol Psychiatr Sci. 2026 Feb 11;35:e10. doi: 10.1017/S2045796026100468 (PMC12925688; doi:10.1017/S2045796026100468)
Supplement: Radha Krishnan et al. supplementary material [file S2045796026100468sup001.docx]

**Supplement 1**

**Antipsychotic treatment patterns and cardiometabolic medicine use:
Current real-world evidence**

Ramya Padmavathy Radha Krishnan^1^, Helga Zoega^2^, Nicholas A Buckley^1^, Jacques E Raubenheimer^1^

^1^Sydney Pharmacy School, Faculty of Medicine and Health, The University of Sydney, Sydney, New South Wales, Australia (R P Radha Krishnan MSc, Prof N A Buckley MD, J E Raubenheimer PhD)

^2^Centre of Public Health Sciences, Faculty of Medicine, University of Iceland, Reykjavik, Iceland (Prof H Zoega PhD)

Corresponding author:

Ramya Padmavathy Radha Krishnan, Sydney Pharmacy School, Faculty of Medicine and Health, RC Mills Building Room 107, The University of Sydney, Sydney, New South Wales 2006, Australia

Email: [ramya.radhakrishnan@sydney.edu.au](mailto:ramya.radhakrishnan@sydney.edu.au)

Table of Contents

[**Supplementary Methods** 3](#_Toc214386339)

[RECORD-PE checklist 3](#_Toc214386340)

[Unsupervised hierarchical clustering 10](#_Toc214386341)

[**Supplementary Tables** 11](#_Toc214386342)

[**eTable 1:** Antipsychotic agents and their PBS item codes 11](#_Toc214386343)

[**eTable 2:** Medicines to treat cardiometabolic comorbidities and their classes based on ATC classification 12](#_Toc214386344)

[**eTable 3:** Antipsychotic treatment patterns between 2017–2022 among antipsychotic-users 16](#_Toc214386345)

[**eTable 4:** Adjusted prevalence ratios for cardiometabolic medicine use with low-dose and continuous use of individual antipsychotic agents 18](#_Toc214386346)

[**eTable 5:** Sensitivity analyses using varying percentiles to estimate treatment average daily dose 19](#_Toc214386347)

[**eTable 6:** Sensitivity analyses using varying percentiles to estimate cumulative treatment duration 20](#_Toc214386348)

[**Supplementary Figures** 21](#_Toc214386349)

[**eFigure 1:** Subject selection flowchart 21](#_Toc214386350)

[**efigure 2:** Unsupervised hierarchical clustering analysis of prevalence ratios for cardiometabolic medicines and antipsychotic pairs 22](#_Toc214386351)

**Supplementary Methods**

## RECORD-PE checklist

| Item No | STROBE items | RECORD items | RECORD-PE items | Page No and/or Explanation |
| --- | --- | --- | --- | --- |
| **Title and abstract** | | | | |
| 1 | (a) Indicate the study’s design with a commonly used term in the title or the abstract.  (b) Provide in the abstract an informative and balanced summary of what was done and what was found. | 1.1: The type of data used should be specified in the title or abstract. When possible, the name of the databases used should be included.  1.2: If applicable, the geographical region and timeframe within which the study took place should be reported in the title or abstract.  1.3: If linkage between databases was conducted for the study, this should be clearly stated in the title or abstract. | — | Abstract Page 2–3 |
| **Introduction** | | | | |
| Background rationale | | | | |
| 2 | Explain the scientific background and rationale for the investigation being reported. | — | — | Introduction page 4–5 |
| Objectives | | | | |
| 3 | State specific objectives, including any prespecified hypotheses. | — | — | Introduction page 5 |
| **Methods** | | | | |
| Study design | | | | |
| 4 | Present key elements of study design early in the paper. | — | 4.a: Include details of the specific study design (and its features) and report the use of multiple designs if used.  4.b: The use of a diagram(s) is recommended to illustrate key aspects of the study design(s), including exposure, washout, lag and observation periods, and covariate definitions as relevant. | Methods page 6  Subject selection flowchart in Supplementary material eFigure 1. |
| Setting | | | | |
| 5 | Describe the setting, locations, and relevant dates, including periods of recruitment, exposure, follow-up, and data collection. | — | — | Methods page 6-8 |
| Participants | | | | |
| 6 | (a) Cohort study—give the eligibility criteria, and the sources and methods of selection of participants. Describe methods of follow-up. Case-control study—give the eligibility criteria, and the sources and methods of case ascertainment and control selection. Give the rationale for the choice of cases and controls. Cross sectional study—give the eligibility criteria, and the sources and methods of selection of participants.  (b) Cohort study—for matched studies, give matching criteria and number of exposed and unexposed. Case-control study—for matched studies, give matching criteria and the number of controls per case. | 6.1: The methods of study population selection (such as codes or algorithms used to identify participants) should be listed in detail. If this is not possible, an explanation should be provided.  6.2: Any validation studies of the codes or algorithms used to select the population should be referenced. If validation was conducted for this study and not published elsewhere, detailed methods and results should be provided.  6.3: If the study involved linkage of databases, consider use of a flow diagram or other graphical display to demonstrate the data linkage process, including the number of individuals with linked data at each stage. | 6.1.a: Describe the study entry criteria and the order in which these criteria were applied to identify the study population. Specify whether only users with a specific indication were included and whether patients were allowed to enter the study population once or if multiple entries were permitted. See explanatory document for guidance related to matched designs. | Methods page 7 |
| Variables | | | | |
| 7 | Clearly define all outcomes, exposures, predictors, potential confounders, and effect modifiers. Give diagnostic criteria, if applicable. | 7.1: A complete list of codes and algorithms used to classify exposures, outcomes, confounders, and effect modifiers should be provided. If these cannot be reported, an explanation should be provided. | 7.1.a: Describe how the drug exposure definition was developed.  7.1.b: Specify the data sources from which drug exposure information for individuals was obtained.  7.1.c: Describe the time window(s) during which an individual is considered exposed to the drug(s). The rationale for selecting a particular time window should be provided. The extent of potential left truncation or left censoring should be specified.  7.1.d: Justify how events are attributed to current, prior, ever, or cumulative drug exposure.  7.1.e: When examining drug dose and risk attribution, describe how current, historical or time on therapy are considered.  7.1.f: Use of any comparator groups should be outlined and justified.  7.1.g: Outline the approach used to handle individuals with more than one relevant drug exposure during the study period. | Methods pages 6–10; Supplementary material eTables 1 and 2 |
| Data sources/measurement | | | | |
| 8 | For each variable of interest, give sources of data and details of methods of assessment (measurement). Describe comparability of assessment methods if there is more than one group. | — | 8.a: Describe the healthcare system and mechanisms for generating the drug exposure records. Specify the care setting in which the drug(s) of interest was prescribed. | Methods pages 6–8 |
| Bias | | | | |
| 9 | Describe any efforts to address potential sources of bias. | — | — | Methods pages 9–10 |
| Study size | | | | |
| 10 | Explain how the study size was arrived at. | — | — | Not applicable, all exposed individuals were included |
| Quantitative variables | | | | |
| 11 | Explain how quantitative variables were handled in the analyses. If applicable, describe which groupings were chosen, and why. | — | — | Methods pages 9–10 |
| Statistical methods | | | | |
| 12 | (a) Describe all statistical methods, including those used to control for confounding.  (b) Describe any methods used to examine subgroups and interactions.  (c) Explain how missing data were addressed.  (d) Cohort study—if applicable, explain how loss to follow-up was addressed. Case-control study—if applicable, explain how matching of cases and controls was addressed. Cross sectional study—if applicable, describe analytical methods taking account of sampling strategy.  (e) Describe any sensitivity analyses. | — | 12.1.a: Describe the methods used to evaluate whether the assumptions have been met.  12.1.b: Describe and justify the use of multiple designs, design features, or analytical approaches. | Methods pages 9–10 |
| Data access and cleaning methods | | | | |
| 12 | — | 12.1: Authors should describe the extent to which the investigators had access to the database population used to create the study population.  12.2: Authors should provide information on the data cleaning methods used in the study. | — | Not applicable, cleaned data provided by Services Australia |
| Linkage | | | | |
| 12 | — | 12.3: State whether the study included person level, institutional level, or other data linkage across two or more databases. The methods of linkage and methods of linkage quality evaluation should be provided. | — | Not applicable, no linkage |
| **Results** | | | | |
| Participants | | | | |
| 13 | (a) Report the numbers of individuals at each stage of the study (eg, numbers potentially eligible, examined for eligibility, confirmed eligible, included in the study, completing follow-up, and analysed).  (b) Give reasons for non-participation at each stage.  (c) Consider use of a flow diagram. | 13.1: Describe in detail the selection of the individuals included in the study (that is, study population selection) including filtering based on data quality, data availability, and linkage. The selection of included individuals can be described in the text or by means of the study flow diagram. | — | Results page 11; Subject selection flowchart in Supplementary material eFigure 1. |
| Descriptive data | | | | |
| 14 | (a) Give characteristics of study participants (eg, demographic, clinical, social) and information on exposures and potential confounders.  (b) Indicate the number of participants with missing data for each variable of interest.  (c) Cohort study—summarise follow-up time (eg, average and total amount). | — | — | Results page 11; Supplementary material eTable 3 |
| Outcome data | | | | |
| 15 | Cohort study—report numbers of outcome events or summary measures over time. Case-control study—report numbers in each exposure category, or summary measures of exposure. Cross sectional study—report numbers of outcome events or summary measures. | — | — | Results page 11; Supplementary material eTable 3 |
| Main results | | | | |
| 16 | (a) Give unadjusted estimates and, if applicable, confounder adjusted estimates and their precision (eg, 95% confidence intervals). Make clear which confounders were adjusted for and why they were included.  (b) Report category boundaries when continuous variables are categorised.  (c) If relevant, consider translating estimates of relative risk into absolute risk for a meaningful time period. | — | — | Results pages 11–14; Table 1 |
| Other analyses | | | | |
| 17 | Report other analyses done—eg, analyses of subgroups and interactions, and sensitivity analyses. | — | — | Results pages 11–14; Table 1, supplementary material eTable 4 |
| **Discussion** | | | | |
| Key results | | | | |
| 18 | Summarise key results with reference to study objectives. | — | — | Discussion page 15 |
| Limitations | | | | |
| 19 | Discuss limitations of the study, taking into account sources of potential bias or imprecision. Discuss both direction and magnitude of any potential bias. | 19.1: Discuss the implications of using data that were not created or collected to answer the specific research question(s). Include discussion of misclassification bias, unmeasured confounding, missing data, and changing eligibility over time, as they pertain to the study being reported. | 19.1.a: Describe the degree to which the chosen database(s) adequately captures the drug exposure(s) of interest. | Discussion page 19 |
| Interpretation | | | | |
| 20 | Give a cautious overall interpretation of results considering objectives, limitations, multiplicity of analyses, results from similar studies, and other relevant evidence. | — | 20.a: Discuss the potential for confounding by indication, contraindication or disease severity or selection bias (healthy adherer/sick stopper) as alternative explanations for the study findings when relevant. | Discussion page 19–20 |
| Generalisability | | | | |
| 21 | Discuss the generalisability (external validity) of the study results. | — | — | Discussion page 18–20 |
| **Other information** | | | | |
| Funding | | | | |
| 22 | Give the source of funding and the role of the funders for the present study and, if applicable, for the original study on which the present article is based. | — | — | Page 22 |
| Accessibility of protocol, raw data, and programming code | | | | |
| 22 | — | 22.1: Authors should provide information on how to access any supplemental information such as the study protocol, raw data, or programming code. | — | Page 23 and supplementary material |

From: Langan SM, Schmidt SA, Wing K, et al. (2018) The reporting of studies conducted using observational routinely collected health data statement for pharmacoepidemiology (RECORD-PE). BMJ: k3532.

## Unsupervised hierarchical clustering

We performed unsupervised hierarchical clustering analyses to identify distinct groups of antipsychotic and cardiometabolic medication pairs based on their co-dispensing patterns. Through this, clustering methods can help us understand medicine pairs that are preferred by clinicians, metabolic risk profiles of individuals and identify areas of concerns, such as drug-drug interactions or unfavourable pairs.

Similar to the main analyses, we first estimated the adjusted prevalence ratios (aPR) for each antipsychotic agent and cardiometabolic medicine pair, comparing antipsychotic-users with non-users and adjusting for age (as a continuous variable) and sex. We then used Ward’s minimum-variance method to derive clusters of antipsychotic-cardiometabolic medicine pairs, to minimise within-cluster variability. We evaluated the resulting clusters through the cubic clustering criterion (CCC) and pseudo F-statistics, which guided the selection of an optimal number of clusters. We also produced a dendrogram to visually represent the cluster hierarchy. Finally, we created a heatmap to represent the aPRs of each antipsychotic agent and cardiometabolic medicine pair, along with the dendrogram to display the results of the clustering analysis. Similarly, we followed the above steps to derive aPR estimates for antipsychotic agent and ATC classes of cardiometabolic medicines.

# **Supplementary Tables**

## **eTable 1:** Antipsychotic agents and their PBS item codes

| **Medicine** | **PBS item codes** |
| --- | --- |
| Amisulpride | 08594H, 08595J, 08596K, 08736T |
| Aripiprazole | 08717T, 08718W, 08719X, 08720Y, 10219W, 10224D |
| Asenapine | 05140M, 05141N |
| Brexpiprazole | 11184P, 11188W, 11189X, 11190Y |
| Cariprazine | 12652X, 12619E, 12622H, 12653Y |
| Chlorpromazine | 01195X, 01196Y, 01197B, 01199D, 01201F |
| Clozapine | 05626D, 05627E, 05628F, 05629G, 05630H, 06101D, 06102E, 06417R, 06418T, 06462D, 06463E, 09632Y, 10288L, 10289M, 10302F, 10341G, 10358E, 11415T, 11422E |
| Flupentixol | 02255Q, 02256R, 02257T |
| Fluphenazine^$^ | 01001Q, 01046C, 03098C |
| Haloperidol | 02761H, 02763K, 02765M, 02766N, 02767P, 02768Q, 02770T |
| Lurasidone | 10526B, 10529E |
| Olanzapine | 01024X, 01037N, 01041T, 01042W, 03381Y, 03382B, 03384D, 03385E, 08170B, 08185T, 08186W, 08187X, 08433W, 08434X, 08952E, 08953F, 09294E, 09295F, 09303P |
| Paliperidone | 05100K, 05102M, 05103N, 05107T, 05109X, 09140C, 09141D, 09142E, 09194X, 11066K, 11072R, 11085K, 11094X, 13046P, 13053B |
| Periciazine | 03052P, 03053Q, 11413Q, 11427K |
| Quetiapine | 05458G, 08456C, 08457D, 08458E, 08580N, 09202H, 09203J, 09204K, 09205L |
| Risperidone | 01842Y, 01846E, 03169T, 03170W, 03171X, 03172Y, 08100H, 08780D, 08781E, 08782F, 08787L, 08788M, 08789N, 08790P, 08791Q, 08792R, 08794W, 08869T, 08870W, 09075P, 09076Q, 09079W, 09080X, 09293D, 11869Q, 11872W, 11873X, 11874Y, 11877D, 11879F, 11881H, 11882J |
| Trifluoperazine^$^ | 02185B, 02186C, 02386N |
| Ziprasidone | 09070J, 09071K, 09072L, 09073M |
| Zuclopenthixol | 08097E |

*Notes:* ^$^ These agents were not dispensed in 2022 due to being discontinued but had minimal dispensings between 2017–2021

Item codes are PBS (Pharmaceutical Benefits Scheme)-specific codes to identify medicines and their dose/formulation.

## **eTable 2:** Medicines to treat cardiometabolic comorbidities and their classes based on ATC classification

| **Class** | **Drug** | **PBS item codes** |
| --- | --- | --- |
| **Dyslipidemia** | | |
| Bile acid sequestrant | Colestyramine | 02967E, 09249T |
| PCSK9 monoclonal antibody | Alirocumab^$^ | 12604J, 12607M |
| PCSK9 monoclonal antibody | Evolocumab | 10958R, 11484K, 11972D, 11977J, 11985T, 11986W |
| Cholesterol absorption inhibitor | Ezetimibe | 08757X, 08881K, 08882L, 09483D, 09484E, 10201X, 10204C, 10207F, 10208G, 10376D, 10377E, 10392Y, 10393B, 11408K |
| Fibrates | Fenofibrate | 09022W, 09023X, 09246P, 09247Q |
| Fibrates | Gemfibrozil | 01453L, 09248R |
| HMGCoA reductase inhibitors | Atorvastatin | 08213G, 08214H, 08215J, 08521L, 09049G, 09050H, 09051J, 09052K, 09053L, 09054M, 09055N, 09056P, 09230T, 09231W, 09232X, 09233Y, 10376D, 10377E, 10392Y, 10393B |
| HMGCoA reductase inhibitors | Fluvastatin | 02863Q |
| HMGCoA reductase inhibitors | Pravastatin | 02833D, 02834E, 08197K, 08829Q, 09237E, 09238F, 09239G, 09240H |
| HMGCoA reductase inhibitors | Rosuvastatin | 02574L, 02584B, 02590H, 02594M, 02606E, 02609H, 02628H, 02636R, 10201X, 10204C, 10207F, 10208G |
| HMGCoA reductase inhibitors | Simvastatin | 02011W, 02012X, 02013Y, 08173E, 08313M, 08881K, 08882L, 09241J, 09242K, 09243L, 09244M, 09245N, 09483D, 09484E |
| **Hyperglycemia** | | |
| Alpha-glucosidase inhibitor | Acarbose | 08188Y, 08189B |
| Biguanide | Metformin | 01801T, 02430X, 03439B, 05474D, 05475E, 05476F, 08607B, 09435N, 09449H, 09450J, 09451K, 10032B, 10033C, 10035E, 10038H, 10044P, 10045Q, 10048W, 10051B, 10055F, 10089B, 10090C, 10510E, 10515K, 10516L, 10626G, 10627H, 10633P, 10639Y, 10640B, 10649L, 10650M, 10677Y, 11270E, 11274J, 11282T, 11285Y, 11294K, 11299Q, 11300R, 11312J, 11313K, 11563N, 11564P, 11566R, 11569X, 11574E, 11580L, 11582N, 11586T |
| DPP4 inhibitors | Alogliptin | 02933J, 02986E, 10032B, 10033C, 10035E |
| DPP4 inhibitors | Linagliptin | 03387G, 10038H, 10044P, 10045Q, 11269D, 11274J, 11280Q, 11282T, 11294K, 11298P, 11303X, 11310G |
| DPP4 inhibitors | Saxagliptin | 08983T, 10048W, 10051B, 10055F, 10128C, 11285Y, 11286B, 11299Q, 11305B, 11311H, 11312J |
| DPP4 inhibitors | Sitagliptin | 09180E, 09181F, 09182G, 09449H, 09450J, 09451K, 10089B, 10090C, 11561L, 11566R, 11572C, 11573D, 11574E, 11576G, 11578J, 11579K, 11580L, 11582N, 11583P, 11586T |
| DPP4 inhibitors | Vildagliptin | 03415R, 05474D, 05475E, 05476F |
| SGLT2 inhibitors | Dapagliflozin | 10011X, 10510E, 10515K, 10516L, 11270E, 11286B, 11291G, 11300R, 11305B, 11313K, 12823X, 13106T |
| SGLT2 inhibitors | Empagliflozin | 10202Y, 10206E, 10626G, 10627H, 10633P, 10639Y, 10640B, 10649L, 10650M, 10677Y, 11269D, 11281R, 11298P, 11303X, 11310G, 11314L, 12918X |
| SGLT2 inhibitors | Ertugliflozin | 11561L, 11563N, 11564P, 11569X, 11577H, 11578J, 11579K, 11583P, 11585R |
| GLP1 analogues | Dulaglutide | 11364D |
| GLP1 analogues | Exenatide | 03423E, 03424F |
| GLP1 analogues | Semaglutide | 12075M, 12080T |
| Insulin analogues | Insulin | 01533Q, 01761Q, 01762R, 01763T, 02062M, 08084L, 08212F, 08390N, 08435Y, 08571D, 08609D, 08874C, 09039R, 09040T, 09224L, 11302W, 11308E, 11417X, 11426J, 11645X, 11706D, 11815W, 12234X, 12236B, 12237C, 12238D, 12254Y, 12255B, 12261H, 12262J, 12268Q |
| Sulfonylureas | Glibenclamide | 02939Q |
| Sulfonylureas | Gliclazide | 02449X, 08535F, 09302N |
| Sulfonylureas | Glimepiride | 08450R, 08451T, 08452W, 08533D |
| Sulfonylureas | Glipizide | 02440K |
| Thiazolidinedione | Pioglitazone | 08694N, 08695P, 08696Q |
| **Hypertension** | | |
| ACE inhibitors | Captopril | 08760C |
| ACE inhibitors | Enalapril | 01368B, 01369C, 01370D, 08477E, 09144G, 09145H |
| ACE inhibitors | Fosinopril | 01182F, 01183G |
| ACE inhibitors | Lisinopril | 02456G, 02457H, 02458J |
| ACE inhibitors | Perindopril | 02190G, 02845R, 03050M, 03051N, 08449Q, 08704D, 09006B, 09007C, 09008D, 09346X, 09347Y, 09348B, 09349C |
| ACE inhibitors | Quinapril | 01969P, 01970Q, 08590D |
| ACE inhibitors | Ramipril | 01316G, 01944H, 01945J, 01946K, 02626F, 02629J, 08470T, 09120B, 09121C, 09122D |
| ACE inhibitors | Trandolapril | 02791X, 02792Y, 02793B, 02857J, 08758Y |
| ARB | Candesartan | 08295N, 08296P, 08297Q, 08504N, 08889W, 09314F, 09315G |
| ARB | Eprosartan | 08624X |
| ARB | Irbesartan | 02136K, 08246B, 08247C, 08248D, 08404H, 08405J |
| ARB | Losartan^$^ | 05452Y, 08203R |
| ARB | Olmesartan | 02147B, 02148C, 02161R, 02166B, 02170F, 02836G, 02864R, 02880N, 02953K, 05292M, 05293N, 05294P, 10005N |
| ARB | Telmisartan | 08355R, 08356T, 08622T, 08623W, 08978M, 08979N, 08980P, 08981Q, 09381R |
| ARB | Valsartan | 05285E, 05286F, 05287G, 05288H, 05289J, 05459H, 05460J, 09368C, 09369D, 09370E, 09371F, 09372G, 09373H, 09374J, 09375K, 09376L, 09377M, 09481B, 09482C |
| Antiadrenergic agents | Methyldopa | 01629R |
| Antiadrenergic agents | Moxonidine | 09019Q, 09020R |
| Beta blockers | Atenolol | 01081X, 02243C |
| Beta blockers | Bisoprolol | 08604W, 08605X, 08606Y |
| Beta blockers | Carvedilol | 08255L, 08256M, 08257N, 08258P |
| Beta blockers | Labetalol | 01566K, 01567L |
| Beta blockers | Metoprolol succinate | 08732N, 08733P, 08734Q, 08735R |
| Beta blockers | Metoprolol tartrate | 01324Q, 01325R |
| Beta blockers | Nebivolol | 09311C, 09312D, 09316H |
| Beta blockers | Oxprenolol^$^ | 02961W |
| Beta blockers | Pindolol^$^ | 03062E |
| Calcium channel blockers | Amlodipine | 02751T, 02752W, 02836G, 02864R, 02880N, 02953K, 05285E, 05286F, 05287G, 05288H, 05289J, 05292M, 05293N, 05294P, 05459H, 05460J, 08978M, 08979N, 08980P, 08981Q, 09049G, 09050H, 09051J, 09052K, 09053L, 09054M, 09055N, 09056P, 09346X, 09347Y, 09348B, 09349C, 09375K, 09376L, 09377M, 10005N |
| Calcium channel blockers | Diltiazem | 01312C, 01313D, 01335G, 08480H |
| Calcium channel blockers | Felodipine | 02361G, 02366M, 02367N, 02626F, 02629J |
| Calcium channel blockers | Lercanidipine | 08534E, 08679T, 09144G, 09145H |
| Calcium channel blockers | Nifedipine | 01906H, 01907J |
| Calcium channel blockers | Verapamil | 01241H, 01250T, 02208F, 02857J |
| Other vasodilators | Hydralazine | 01639G, 01640H |
| Other vasodilators | Minoxidil | 02313R |
| Potassium-sparing diuretic | Amiloride | 01486F |
| Sulfonamide diuretics | Chlortalidone | 01585K |
| Sulfonamide diuretics | Indapamide | 02190G, 02436F, 02845R, 08449Q, 08532C |
| Thiazide diuretic | Hydrochlorothiazide | 01484D, 01486F, 02136K, 02161R, 02166B, 02170F, 02836G, 02864R, 02880N, 02953K, 05285E, 05286F, 05287G, 05288H, 05289J, 08404H, 08405J, 08477E, 08504N, 08590D, 08622T, 08623W, 08624X, 09314F, 09315G, 09372G, 09373H, 09374J, 09381R, 09481B, 09482C, 10005N |
| **Thrombosis** | | |
| Factor Xa inhibitors | Apixaban | 02735Y, 02744K, 05054B, 05061J, 10414D |
| Factor Xa inhibitors | Rivaroxaban | 02160Q, 02268J, 02691P, 09466F, 09467G, 09469J, 11633G, 12192Q |
| Factor Xa inhibitors | Fondaparinux^$^ | 08775W |
| Platelet aggregation inhibitors | Aspirin | 08202Q, 09296G |
| Platelet aggregation inhibitors | Clopidogrel | 02275R, 08358X, 09296G, 09317J, 09354H |
| Platelet aggregation inhibitors | Dipyridamole^$^ | 08335Q |
| Platelet aggregation inhibitors | Eptifibatide^$^ | 08683B, 08684C |
| Platelet aggregation inhibitors | Ticagrelor | 01418P |
| Direct thrombin inhibitors | Dabigatran | 02753X, 02769R, 09321N |
| Direct thrombin inhibitors | Bivalirudin^$^ | 08844L |
| Heparins | Enoxaparin | 05434B, 05435C, 08262W, 08263X, 08264Y, 08510X, 08558K, 08639Q, 08640R, 08716R |
| Heparins | Heparin | 01463B, 01466E |
| Vitamin K antagonist | Warfarin | 02209G, 02211J, 02843P, 02844Q |

*Notes:*

ACE: Angiotensin-converting enzyme, ARB: Angiotensin receptor blockers, DPP4: Dipeptidyl peptidase 4, GLP1: Glucagon-like peptide 1, HMGCoA: Hydroxymethylglutaryl-CoA, PCSK9: Proprotein convertase subtilisin/kexin type 9, SGLT2: Sodium-glucose transport protein 2

^$^These medicines were excluded from the individual analysis due to low number of users.

Notes: Other cardiovascular medicine classes such as drugs for heart failure (aldosterone antagonists including spironolactone and eplerenone, loop diuretics, sympathomimetics), anti-angina medicines, anti-arrhythmics, drugs for pulmonary hypertension (prostacyclins, endothelin antagonists, phosphodiesterase-5 inhibitors) and drugs for peripheral vascular disease are not included in this study.

## **eTable 3:** Antipsychotic treatment patterns between 2017–2022 among antipsychotic-users

|  | N | | % |
| --- | --- | --- | --- |
| Average daily dose (SD), mg/day | 9.6 (8.4) | |  |
| Average daily dose categories  Low (>0–5.0 mg/day)  Moderate (>5.0–10.0 mg/day)  High (>10.0–20.0 mg/day)  Very high (>20 mg/day) | 9,731  7,985  7,627  3,002 | | 34.3  28.2  26.9  10.6 |
| Treatment duration in days, median (IQR) | 1,420 (592–1,976) | |  |
| Treatment duration categories  Short-term (≤6 months)  Medium-term (>6 months–1 year)  Long-term (>1–3 years)  Ultra long-term (>3 years) | 2,241  2,485  6,700  16,919 | | 7.9  8.8  23.6  59.7 |
| Antipsychotic polypharmacy | 12,387 | | 32.5 |
| Low-dose continuous treatment (≥1 year) | 9,082 | | 27.8 |
| Antipsychotic agent, overall (monotherapy) ^$^  Amisulpride  Aripiprazole  Asenapine  Brexpiprazole  Cariprazine†  Chlorpromazine  Clozapine  Flupenthixol  Fluphenazine†  Haloperidol  Lurasidone  Olanzapine  Paliperidone  Periciazine  Quetiapine  Risperidone  Trifluoperazine†  Ziprasidone  Zuclopenthixol | 28345  1,460  6,511  737  1,262  147  1,649  1,825  370  49  899  2,518  11,410  3,619  1,138  14,389  5,415  13  525  1,104 | (18,106)  (299)  (2,052)  (166)  (287)  (20)  (514)  (532)  (63)  (5)  (260)  (610)  (5,143)  (999)  (547)  (8,376)  (2,594)  (5)  (132)  (200) | (63.9)  5.2 (20.5)  23 (31.5)  2.6 (22.5)  4.5 (22.7)  0.5 (13.6)  5.8 (31.2)  6.4 (29.2)  1.3 (17)  0.2 (10.2)  3.2 (28.9)  8.9 (24.2)  40.3 (45.1)  12.8 (27.6)  4 (48.1)  50.8 (58.2)  19.1 (47.9)  0 (38.5)  1.9 (25.1)  3.9 (18.1) |

*Notes:* ^$^ overall includes both monotherapy and antipsychotic polypharmacy.
% in parentheses indicates the percentage of monotherapy out of overall subjects for that agent. Individuals could contribute to multiple monotherapy periods due to antipsychotic switching.
† These antipsychotics were excluded in the individual analyses by antipsychotic agent due to low number of users.

## **eTable 4:** Adjusted prevalence ratios for cardiometabolic medicine use with low-dose and continuous use of individual antipsychotic agents

| Antipsychotic | Number of individuals | Any cardiometabolic medicine use | Lipid-modifying agent use | Anti-diabetic use | Anti-hypertensive use | Anti-thrombotic use |
| --- | --- | --- | --- | --- | --- | --- |
| Amisulpride | 126 | 1.28 (0.97–1.68) | 1.15 (0.75–1.74) | **2.44 (1.65–3.62)** | 1.04 (0.71–1.52) | 0.83 (0.31–2.21) |
| Aripiprazole | 523 | **1.39 (1.19–1.63)** | **1.36 (1.07–1.73)** | **1.77 (1.36–2.30)** | 1.09 (0.87–1.36) | 1.03 (0.62–1.72) |
| Asenapine | 169 | **1.32 (1.01–1.71)** | 1.45 (1.00–2.09) | **1.82 (1.18–2.78)** | 1.29 (0.93–1.80) | 0.93 (0.39–2.25) |
| Brexpiprazole | 156 | **1.43 (1.09–1.88)** | 1.45 (0.97–2.19) | **1.64 (1.01–2.68)** | 1.02 (0.68–1.54) | 0.89 (0.33–2.38) |
| Chlorpromazine | 387 | **1.34 (1.14–1.57)** | **1.43 (1.14–1.78)** | **2.23 (1.75–2.83)** | 1.16 (0.94–1.43) | **1.92 (1.32–2.80)** |
| Haloperidol | 162 | 1.06 (0.82–1.37) | 1.15 (0.83–1.62) | **1.23 (0.76–1.98)** | 0.86 (0.61–1.21) | 1.66 (0.92–3.01) |
| Lurasidone | 233 | **1.39 (1.11–1.75)** | 1.12 (0.76–1.65) | **1.70 (1.15–2.52)** | 1.29 (0.96–1.74) | 0.89 (0.40–1.99) |
| Olanzapine | 1342 | **1.13 (1.03–1.25)** | **1.25 (1.10–1.43)** | **1.33 (1.12–1.58)** | 1.04 (0.92–1.18) | 1.16 (0.89–1.52) |
| Periciazine | 500 | **1.28 (1.11–1.48)** | **1.44 (1.18–1.74)** | **1.87 (1.48–2.35)** | 1.19 (0.99–1.43) | **1.54 (1.06–2.23)** |
| Quetiapine | 4508 | **1.27 (1.20–1.33)** | **1.43 (1.34–1.54)** | **1.49 (1.36–1.63)** | **1.11 (1.04–1.19)** | **1.54 (1.35–1.76)** |
| Risperidone | 1441 | 1.09 (0.98–1.22) | **1.25 (1.08–1.45)** | **1.60 (1.34–1.92)** | 0.92 (0.80–1.07) | 1.07 (0.78–1.48) |

*Notes:* Prevalence ratios (adjusted for age and sex) for cardiometabolic medicine use, comparing antipsychotic-users with non-users, for low-dose antipsychotic use lasting ≥1 year. Analyses were run for antipsychotic agents used by 100 or more individuals.

## **eTable 5:** Sensitivity analyses using varying percentiles to estimate treatment average daily dose

| **Outcome** | **ADD Categories** | **aPR (95% CI)** | | | | |
| --- | --- | --- | --- | --- | --- | --- |
|  |  | **p70** | **p75** | **p80** | **p85** | **p90** |
| Any use | Low | 1.220 (1.176-1.266) | 1.230 (1.186-1.276) | **1.227 (1.183-1.272)** | 1.228 (1.185-1.273) | 1.226 (1.184-1.270) |
|  | Moderate | 1.279 (1.232-1.327) | 1.267 (1.220-1.315) | **1.276 (1.229-1.324)** | 1.276 (1.230-1.325) | 1.272 (1.225-1.320) |
|  | High | 1.363 (1.315-1.413) | 1.369 (1.320-1.419) | **1.366 (1.317-1.417)** | 1.371 (1.322-1.423) | 1.386 (1.336-1.439) |
|  | Very high | 1.528 (1.453-1.607) | 1.534 (1.458-1.615) | **1.537 (1.460-1.618)** | 1.534 (1.457-1.616) | 1.535 (1.457-1.618) |
| Lipid modifiers | Low | 1.355 (1.287-1.427) | 1.365 (1.297-1.436) | **1.354 (1.287-1.424)** | 1.362 (1.296-1.432) | 1.369 (1.304-1.438) |
|  | Moderate | 1.455 (1.382-1.530) | 1.444 (1.373-1.519) | **1.462 (1.390-1.538)** | 1.451 (1.379-1.527) | 1.453 (1.381-1.528) |
|  | High | 1.658 (1.582-1.738) | 1.666 (1.588-1.747) | **1.671 (1.593-1.753)** | 1.688 (1.609-1.772) | 1.695 (1.614-1.780) |
|  | Very high | 1.962 (1.841-2.092) | 1.977 (1.854-2.108) | **1.971 (1.847-2.103)** | 1.963 (1.839-2.096) | 1.962 (1.837-2.097) |
| Anti-diabetics | Low | 1.554 (1.459-1.655) | 1.558 (1.464-1.658) | **1.559 (1.466-1.658)** | 1.558 (1.466-1.655) | 1.557 (1.467-1.653) |
|  | Moderate | 1.970 (1.859-2.088) | 1.961 (1.851-2.078) | **1.985 (1.874-2.103)** | 2.004 (1.892-2.122) | 2.006 (1.893-2.124) |
|  | High | 2.495 (2.371-2.627) | 2.519 (2.392-2.652) | **2.518 (2.390-2.652)** | 2.538 (2.408-2.675) | 2.581 (2.448-2.721) |
|  | Very high | 3.054 (2.847-3.276) | 3.100 (2.888-3.326) | **3.111 (2.898-3.340)** | 3.112 (2.896-3.343) | 3.129 (2.910-3.364) |
| Anti-hypertensives | Low | 1.076 (1.025-1.131) | 1.084 (1.032-1.138) | **1.080 (1.030-1.133)** | 1.078 (1.028-1.131) | 1.072 (1.023-1.124) |
|  | Moderate | 1.033 (0.982-1.088) | 1.019 (0.968-1.073) | **1.023 (0.972-1.077)** | 1.024 (0.973-1.078) | 1.023 (0.972-1.077) |
|  | High | 1.058 (1.006-1.112) | 1.058 (1.006-1.114) | **1.061 (1.008-1.117)** | 1.060 (1.006-1.116) | 1.070 (1.016-1.128) |
|  | Very high | 1.084 (1.007-1.167) | 1.098 (1.019-1.182) | **1.089 (1.010-1.174)** | 1.094 (1.014-1.180) | 1.089 (1.009-1.176) |
| Anti-thrombotics | Low | 1.507 (1.371-1.657) | 1.512 (1.378-1.660) | **1.521 (1.388-1.668)** | 1.542 (1.410-1.687) | 1.537 (1.407-1.679) |
|  | Moderate | 1.363 (1.231-1.510) | 1.345 (1.214-1.491) | **1.313 (1.183-1.456)** | 1.310 (1.181-1.454) | 1.315 (1.185-1.458) |
|  | High | 1.349 (1.219-1.493) | 1.375 (1.242-1.522) | **1.404 (1.268-1.554)** | 1.375 (1.239-1.526) | 1.366 (1.229-1.519) |
|  | Very high | 1.583 (1.374-1.823) | 1.547 (1.338-1.787) | **1.527 (1.319-1.768)** | 1.535 (1.324-1.779) | 1.547 (1.332-1.795) |

*Notes:* Sensitivity analyses were run using varying percentiles (p75 to p90) to estimate the days covered per unit of the antipsychotic formulation. p80 was chosen as the cut-off percentile for the main analyses.

ADD: average daily dose, aPR: adjusted prevalence ratio, CI: confidence interval

## **eTable 6:** Sensitivity analyses using varying percentiles to estimate cumulative treatment duration

| **Outcome** | **Duration Categories** | **aPR (95% CI)** | | | | |
| --- | --- | --- | --- | --- | --- | --- |
|  |  | **p70** | **p75** | **p80** | **p85** | **p90** |
| Any use | Short-term | 1.132 (1.041-1.230) | 1.134 (1.040-1.237) | **1.117 (1.021-1.221)** | 1.112 (1.013-1.220) | 1.117 (1.012-1.233) |
|  | Medium-term | 1.132 (1.044-1.227) | 1.134 (1.047-1.229) | **1.137 (1.049-1.233)** | 1.131 (1.043-1.226) | 1.136 (1.047-1.232) |
|  | Long-term | 1.235 (1.181-1.291) | 1.236 (1.182-1.294) | **1.240 (1.184-1.297)** | 1.247 (1.192-1.306) | 1.241 (1.184-1.300) |
|  | Ultra long-term | 1.380 (1.347-1.413) | 1.376 (1.344-1.409) | **1.374 (1.343-1.407)** | 1.371 (1.339-1.403) | 1.369 (1.337-1.401) |
| Lipid modifiers | Short-term | 1.219 (1.079-1.378) | 1.214 (1.069-1.379) | **1.200 (1.053-1.368)** | 1.180 (1.029-1.354) | 1.143 (0.985-1.326) |
|  | Medium-term | 1.304 (1.164-1.460) | 1.310 (1.171-1.466) | **1.302 (1.162-1.458)** | 1.319 (1.178-1.477) | 1.325 (1.183-1.484) |
|  | Long-term | 1.297 (1.215-1.384) | 1.292 (1.209-1.380) | **1.295 (1.212-1.385)** | 1.303 (1.219-1.394) | 1.306 (1.220-1.398) |
|  | Ultra long-term | 1.675 (1.624-1.727) | 1.671 (1.620-1.722) | **1.668 (1.618-1.719)** | 1.661 (1.611-1.712) | 1.655 (1.606-1.706) |
| Anti-diabetics | Short-term | 1.417 (1.234-1.628) | 1.428 (1.237-1.648) | **1.369 (1.179-1.590)** | 1.352 (1.157-1.581) | 1.329 (1.124-1.571) |
|  | Medium-term | 1.328 (1.156-1.527) | 1.341 (1.168-1.540) | **1.371 (1.194-1.573)** | 1.355 (1.180-1.557) | 1.411 (1.231-1.618) |
|  | Long-term | 1.703 (1.585-1.830) | 1.694 (1.575-1.822) | **1.693 (1.573-1.822)** | 1.709 (1.588-1.840) | 1.665 (1.544-1.795) |
|  | Ultra long-term | 2.436 (2.352-2.523) | 2.424 (2.341-2.511) | **2.418 (2.335-2.503)** | 2.405 (2.323-2.490) | 2.398 (2.317-2.482) |
| Anti-hypertensives | Short-term | 0.996 (0.889-1.115) | 0.992 (0.882-1.117) | **0.992 (0.879-1.120)** | 0.986 (0.869-1.118) | 1.010 (0.885-1.154) |
|  | Medium-term | 0.998 (0.895-1.113) | 1.015 (0.912-1.130) | **1.012 (0.908-1.128)** | 1.011 (0.907-1.127) | 1.018 (0.913-1.136) |
|  | Long-term | 1.048 (0.986-1.114) | 1.045 (0.982-1.112) | **1.040 (0.977-1.107)** | 1.042 (0.978-1.110) | 1.029 (0.965-1.098) |
|  | Ultra long-term | 1.075 (1.040-1.111) | 1.074 (1.039-1.109) | **1.075 (1.040-1.110)** | 1.074 (1.040-1.109) | 1.074 (1.040-1.109) |
| Anti-thrombotics | Short-term | 1.704 (1.410-2.058) | 1.737 (1.430-2.110) | **1.687 (1.379-2.063)** | 1.699 (1.380-2.092) | 1.732 (1.391-2.157) |
|  | Medium-term | 1.433 (1.174-1.750) | 1.438 (1.179-1.753) | **1.447 (1.185-1.766)** | 1.498 (1.231-1.823) | 1.439 (1.176-1.760) |
|  | Long-term | 1.443 (1.285-1.620) | 1.446 (1.286-1.625) | **1.473 (1.311-1.656)** | 1.473 (1.309-1.657) | 1.496 (1.329-1.683) |
|  | Ultra long-term | 1.398 (1.308-1.495) | 1.396 (1.307-1.492) | **1.393 (1.304-1.488)** | 1.389 (1.301-1.484) | 1.390 (1.302-1.484) |

*Notes:* Sensitivity analyses were run using varying percentiles (p75 to p90) to estimate the days covered per unit of the antipsychotic formulation. p80 was chosen as the cut-off percentile for the main analyses.

aPR: adjusted prevalence ratio, CI: confidence interval

# **Supplementary Figures**

Individuals with any PBS dispensing in 2022

(N=1,741,076)

At least one antipsychotic dispensing in 2022

(N=46,746)

No antipsychotic dispensing in 2022

(N=1,694,330)

AP-users with at least two antipsychotic dispensings in 2022

(N=28,345)

Excluded individuals

i) only one oral antipsychotic dispensing in 2022 (N=7,253)

ii) age <15 or >64 in 2022 (N=11,148)

Excluded individuals

i) with previous antipsychotic dispensings (N=57,510)

ii) age <15 or >64 in 2022 (N=620,210)

Non-users with no previous antipsychotic dispensings

(N=1,016,610)

## **eFigure 1:** Subject selection flowchart

## **
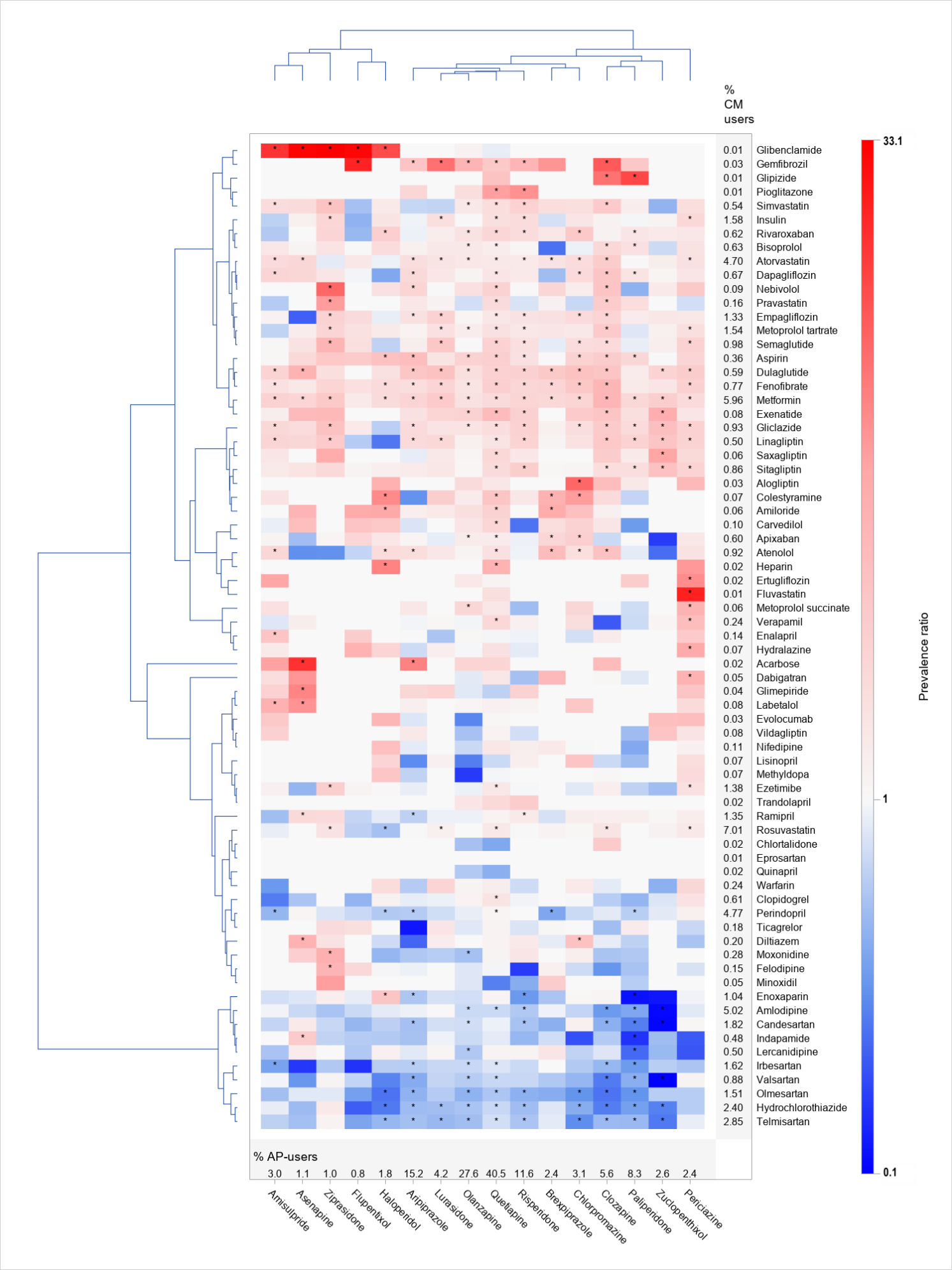
efigure 2:** Unsupervised hierarchical clustering analysis of prevalence ratios for cardiometabolic medicines and antipsychotic pairs

*Notes*: Unsupervised hierarchical clustering analysis using the log-transformed prevalence ratios (adjusted for age and sex) comparing antipsychotic-users with non-users for each pairwise combination of antipsychotic and cardiometabolic medicine. Negative values (blue gradient) are prevalent among non-users and positive values (red gradient) are prevalent among AP-users. Significant 95% confidence intervals are indicated by an asterisk (*). The percentage of individuals using that cardiometabolic medicine or antipsychotic is given. Cardiometabolic medicines with <0.01% of users were not included in the analysis (alirocumab, oxprenolol, captopril, losartan, fosinopril, bivalirudin, fondaparinux, dipyridamole, and eptifibatide). CM: cardiometabolic medicine, AP: antipsychotic
